# Unilateral Ovarian Abscess Caused by *Salmonella*

**DOI:** 10.1080/10647440300025524

**Published:** 2003

**Authors:** Toshimitsu Tohya, Toshihiro Yoshimura, Chikashi Onoda

**Affiliations:** Department of Obstetrics and Gynecology Kumamoto Rosai Hospital Yatsushiro Kumamoto 866-8533 Japan

## Abstract

*Background:* Patients with unilateral ovarian abscesses due to *Salmonella* are rare.

*Case report:* A 48-year-old woman with a left ovarian abscess caused by *Salmonella*  group O7 is reported.

*Conclusion:*  In our patient, the ovary may have been seeded hematogenously by *salmonellae*  and may have evolved into a local infection.


*Infect Dis Obstet Gynecol 2003;11:217–219*

### Unilateral ovarian abscess caused by *Salmonella*

**Toshimitsu Tohya, Toshihiro Yoshimura and Chikashi Onoda**

*Department of Obstetrics and Gynecology, Kumamoto Rosai Hospital, Yatsushiro, Kumamoto, Japan*

***Background:*** Patients with unilateral ovarian abscesses due to *Salmonella* are rare.
***Case report:*** A 48-year-old woman with a left ovarian abscess caused by *Salmonella* group O7 is reported.
***Conclusion:*** In our patient, the ovary may have been seeded hematogenously by salmonellae and may have evolved into a local infection.

Key words: TUBO-OVARIAN ABSCESS; PELVIC INFLAMMATORY DISEASE; HEMATOGENOUS INFECTION

### INTRODUCTION

Ovarian abscesses due to *Salmonella* infection are rare. In 6250 cases of salmonellosis treated at one hospital in India, only one case of *Salmonella* ovarian abscess was reported[1]. Between 1977 and 1983, Cohen and co-workers[2] reported 8000 *Salmonella* infections at Duke University Medical Center, NC, USA and only nine cases of ovarian abscess were included. We report a case of ovarian abscess caused by *Salmonella* O7 (nontyphoid *Salmonella*) and discuss the route of infection.

### CASE REPORT

A 48-year-old woman, gravida 3, para 3, had a left ovarian cyst 45 mm in diameter as diagnosed by transvaginal ultrasonography and had been followed for 1 year in our institution. She attended another clinic complaining of lower abdominal pain and chills. She had a fever of 39°C and treatment with intravenous piperacillin was instituted. Her abdominal pain persisted, but without diarrhea and 18 days later she was transferred to our hospital. Manual palpation revealed tenderness in the lower left quadrant of the abdomen, above the uterine cervix. Ultrasound examination revealed that the ovarian mass was cystic and measured $54 \times 62 \times 64$ mm in size. Computed tomography scanning revealed that the lesion was inhomogeneous, which became more obvious after an intravenous bolus of contrast material enhanced the image of the central, low-density region of the mass. Intravenous pyelography showed no abnormal findings. Initial laboratory evaluation revealed a hemoglobin count of 9.3 g/dl and a white cell count of 11 000/μl. The C-reactive protein level was 16.8 mg/dl. Tumor markers were significantly elevated (CA125: 314 u/ml, CA19-9: 72 u/ml). Papanicolaou smear tests of the uterine cervix and endometrium were negative. Blood culture was also negative.

The patient was initially managed with antibiotic (cefotiam) therapy. However, the lower abdominal pain persisted and the tender mass became more obvious. A laparotomy was performed 6 days after admission. The laparotomy

Correspondence to: Toshimitsu Tohya, MD, Department of Obstetrics and Gynecology, Kumamoto Rosai Hospital, Yatsushiro, Kumamoto, 866-8533, Japan. Email: tohya@fine.ocn.ne.jp

© 2003 The Parthenon Publishing Group 

revealed an unruptured left ovarian abscess adherent to the sigmoid colon and the uterus. The left oviduct was not affected. The uterus, right ovary and right fallopian tube were intact. There was no evidence of diverticulitis or appendicitis. Subsequently, bilateral salpingo-oophorectomy and total hysterectomy with transabdominal drainage were performed. The ovary showed no histologic neoplastic change and the oviducts had only slight inflammatory change. There was no inflammatory change in the endometrium. The patient recovery was uneventful.

On bacteriological culture, the exudate from the left ovarian abscess yielded heavy growth of *Salmonella* O7. A postoperative stool culture also grew the same organism. Gonorrhea culture was negative. The patient was then treated with a 7-day course of intravenous sulbactam/cefoperazone (1 g two times a day). Four weeks after surgery, she exhibited no symptoms and pelvic examination was normal. However, 1 month postoperatively, follow-up stool examination was positive for *Salmonella*. Therefore she received oral levofloxacin (100 mg, 3 times a day for 14 days). Stool culture was repeated and approximately 6 months later was negative for *Salmonella* O7.

### DISCUSSION

We initially thought that this *Salmonella* infection was a case of pelvic inflammatory disease (PID), which is caused by microorganisms colonizing the endocervix and ascending to the endometrium and fallopian tubes. There are case reports of *Salmonella* bilateral salpingo-oophoritis associated with *Chlamydia* infection suggestive of sexually transmitted diseases (STDs)[3]. However, the pathophysiology of *Salmonella* infection is more complex. In the female genital tract, the main route of this infection may be hematogenous spread or through direct contact with the inflamed bowel wall and may involve the ascending route.

This patient's endometrium and fallopian tubes did not undergo macroscopic or microscopic inflammatory change and the ipsilateral ovary was not affected. Therefore, a purulent inflammatory process secondary to the passage of bacteria from the uterine cavity into the tubal lumen is not likely.

*Salmonella* translocate across the mucosa of the small intestine to local or regional tissues and can be found in mesenteric lymph nodes[4]. Any anatomical site may be seeded hematogenously by salmonellae and may evolve into a local infection[5]. The ovary is a rare site for such local infections[2]. Survival within phagocytic cells is essential for *Salmonella* virulence. *Salmonella* proliferates within macrophages and avoids phagocytosis by neutrophils to establish a systemic infection[6,7], thus providing a means of extraintestinal dissemination.

Hematogenously disseminated *Salmonella* tend to localize in sites of pre-existing disease; almost all reported cases of isolated ovarian abscesses had ovarian abnormalities such as a dermoid cyst, endometrioma, cystadenoma or simple cyst as the predisposing factor[2]. In reviewing the 34 cases of ovarian abscess attributed to *Salmonella typhi* in the literature, Cohen and colleagues[2] reported that, in most cases, an isolated ovarian abscess invariably occurs, unlike other ovarian abscesses usually associated with salpingitis. They also stated that there is often a long delay of several weeks between the occurrence of acute typhoid fever and the appearance of an ovarian abscess. A 13-year-old girl with no history of sexual activity[8] and a 16-year-old girl who had a few different, condom-protected sexual contacts[9] each had an ovarian abscess caused by *Salmonella*. These findings strongly suggest that ovarian abscesses due to *Salmonella* infection are hematogenously disseminated.

The present report describes an unusual microbiological agent of pelvic infection. This suggests that gastrointestinal pathogens should be considered as potential etiologic organisms in patients presenting with signs of PID.

Some clinicians recommend conservative management of these cases. However, early surgical intervention is necessary in some patients, especially women of older reproductive age who are at lower risk for STDs and who are more likely to have infections secondary to other surgically treated conditions such as diverticular disease and genital or extragenital cancer.

### REFERENCES

1. Lalitha MK, John R. Unusual manifestation of salmonellosis – a surgical problem. *Q J Med* 1994; 87:301–9
2. Cohen JI, Bartlett JA, Corey RC. Extra-intestinal manifestations of *salmonella* infections. *Medicine* 1987;66:349–88
3. Kostiala AA, Ranta T. Pelvic inflammatory disease caused *Salmonella panama* and its treatment with ciprofloxacin. *Br J Obstet Gynaecol* 1989;96:120–2
4. Runkel NS, Rodriguez LF, Moody FG, *et al.* *Salmonella* infection of the biliary and intestinal tract of wild oppossums. *Lab Anim Sci* 1991;41:54–6
5. Hohmann EL. Nontyphoidal salmonellosis. *Clin Infect Dis* 2001;32:263–9
6. Jepson MA, Clark MA. The role of M cells in *Salmonella* infection. *Microbes Infect* 2001;3: 1183–90
7. Vazquez-Torres A, Fang FC. Cellular routes of invasion by enteropathogens. *Curr Opin Microbiol* 2000;3:54–9
8. Chiva LM, Ergeneli M, Santisteban J. *Salmonella* abscess of the ovary. *Am J Obstet Gynecol* 1995; 172:215–16
9. Burgmans JPJ, van Erp EJM, Brimicombe RW, Kazzaz BA. *Salmonella* enteritidis in an endometriotic ovarian cyst. *Eur J Obstet Gynecol Reprod Biol* 1997;72:207–11

Received 02/21/03; Accepted 07/16/03

## References

[PDF_00196] Burgmans J. P., van Erp E. J., Brimicombe R. W., Kazzaz B. A. (1997). Salmonella enteritidis in an endometriotic ovarian cyst.. Eur J Obstet Gynecol Reprod Biol.

[PDF_00193] Chiva L. M., Ergeneli M., Santisteban J. (1995). Salmonella abscess of the ovary.. Am J Obstet Gynecol.

[PDF_00176] Cohen J. I., Bartlett J. A., Corey G. R. (1987). Extra-intestinal manifestations of salmonella infections.. Medicine (Baltimore).

[PDF_00185] Hohmann E. L. (2001). Nontyphoidal salmonellosis.. Clin Infect Dis.

[PDF_00187] Jepson M. A., Clark M. A. (2001). The role of M cells in Salmonella infection.. Microbes Infect.

[PDF_00179] Kostiala A. A., Ranta T. (1989). Pelvic inflammatory disease caused by Salmonella panama and its treatment with ciprofloxacin. Case report.. Br J Obstet Gynaecol.

[PDF_00173] Lalitha M. K., John R. (1994). Unusual manifestations of salmonellosis--a surgical problem.. Q J Med.

[PDF_00182] Runkel N. S., Rodriguez L. F., Moody F. G., LaRocco M. T., Blasdel T. (1991). Salmonella infection of the biliary and intestinal tract of wild opossums.. Lab Anim Sci.

[PDF_00190] Vazquez-Torres A., Fang F. C. (2000). Cellular routes of invasion by enteropathogens.. Curr Opin Microbiol.

